# Are E-Cigarette and Tea Cigarette Gifting Behaviors Associated with Tobacco Use and Failed Quit Attempts in China?

**DOI:** 10.3390/ijerph192215333

**Published:** 2022-11-20

**Authors:** Huan Zhou, Connie Hoe, Weifang Zhang, Xiaozhao Yang, Mingyan Li, Dan Wu

**Affiliations:** 1 Stomatology Hospital, School of Stomatology, Zhejiang University School of Medicine, Zhejiang Provincial Clinical Research Center for Oral Diseases, Key Laboratory of Oral Biomedical Research of Zhejiang Province, Cancer Center of Zhejiang University, Hangzhou 310006, China; 2 Bloomberg School of Public Health, Johns Hopkins University, Baltimore, MD 21205, USA; 3 Heidelberg Institute of Global Health, Heidelberg University, 69120 Heidelberg, Germany; 4 Department of Sociology and Social Work, Sun Yat-sen University, Guangzhou 510275, China; 5 School of Psychology, Shenzhen Humanities & Social Sciences Key Research Bases of the Center for Mental Health, Shenzhen University, Shenzhen 518060, China; 6 Department of Psychology/Research Center for Quality of Life and Applied Psychology, Guangdong Medical University, Dongguan 523808, China

**Keywords:** e-cigarette, tea cigarette, social participation, cigarette gifting

## Abstract

This study aims to investigate e-cigarette and tea cigarette gifting in China and their influencing factors, as well as to explore whether they were associated with tobacco use and cessation. Using a multistage sampling design, 1512 household heads from Guangdong and Shaanxi provinces were recruited for the study and filled out an online questionnaire about smoking status, social participation, e-cigarette, and tea cigarette gifting. Results showed that more than 30% and nearly 3% of participants had been gifted tea cigarettes and e-cigarettes, respectively. Marital status, province of residence, smoking status, and social participation were associated with gifting behaviors. Logistic regressions showed that receiving e-cigarettes (*OR* = 3.43, *p* < 0.05) and tea cigarettes (*OR* = 1.70, *p* < 0.01) were related to tobacco use. Smokers who have received e-cigarettes (*OR* = 9.85, *p* < 0.01) and tea cigarettes (*OR* = 1.92, *p* < 0.05) were also less likely to quit smoking.

## 1. Introduction

Tobacco use is a major public health concern; over 8 million people are killed by tobacco-related diseases each year. Tobacco use also costs the global economy 1.4 trillion dollars annually [1]. The global public health community has made great strides in reducing tobacco use [2]. However, the tobacco industry has continued to resist the policy measures and aggressively market new products such as electronic cigarettes (e-cigarettes) or electronic nicotine delivery systems (ENDS) and nicotine pouches; these new products have proliferated in many countries [3].

Among all cigarette substitute products, electronic cigarettes (e-cigarettes) have become increasingly popular, and their use has increased worldwide [4]. Their use has also been encouraged by some as replacements for traditional tobacco as they release fewer toxic substances [5,6]. Some studies have shown the efficacy and safety of e-cigarettes in helping smokers reduce tobacco consumption, and it has the same effectiveness as nicotine patches [7,8]. On the other hand, however, research has also demonstrated that e-cigarettes produce toxic chemicals and are potentially harmful to health [9,10,11]. E-cigarette use has also been found to be a risk factor for cigarette smoking initiation and failure in quit attempts [12,13,14]. Further, studies have also shown that e-cigarette use introduces adolescents and young adults to cigarette smoking-related behavior and leads to nicotine addiction [15].

According to the Chinese Adult Tobacco Consumption Survey, the prevalence of e-cigarette smoking in China increased from 1.3% to 1.6% within three years [16,17]. The Chinese government issued the “Notice on Further Protecting Minors from Electronic Cigarettes” in 2019, which banned e-cigarette advertising and the selling of e-cigarettes on e-commerce platforms and websites [18]. Then on 11 March 2022, China’s State Tobacco Monopoly Administration issued a regulation that aims to strengthen the oversight of electronic cigarettes [19]. This regulation, however, does not ban in-store sales or e-cigarette use.

As a result of the popularity of e-cigarettes and the banning of their online sales, a new kind of cigarette substitute has recently emerged on the Chinese market, which is called the “tea cigarette”. It uses different varieties of tea as the main raw material and has different flavors. It is shaped like a traditional cigarette but without tobacco [20]. After being lit, the tea is smoked. Tea cigarette is actually “smoking tea” [20]. This new cigarette substitute has been promoted by its manufacturers as a healthier alternative to smoking [4]. The manufacturers of tea cigarettes are mainly tea/technology companies. In January 2020, the online sales of tea cigarettes reached 6.8 million dollars per month [20], and the offline sales of tea cigarettes remain unknown yet. Unfortunately, the health effects of tea cigarettes are still unknown [21]. Since the main raw material is tea, tea cigarettes have not yet been included in the tobacco regulatory scope. As such, the production and sales of tea cigarettes in China have become a regulatory challenge in the country [22]. Given this, there is an urgent need to conduct research in this area to provide scientific evidence for policymakers to facilitate their decision-making.

A key risk factor for tobacco use in China is the social norm of gifting cigarettes [23]. Cigarette gifting means offering and accepting single cigarettes for immediate consumption in a wide variety of social settings [24]. It is one of the main causes of smoking [25] and contributes to the failure of cessation [26]. A cross-sectional survey in China showed that offering cigarettes is significantly associated with tobacco use, and those who had received cigarettes were 2.58 times more likely to be current smokers than those who did not receive cigarettes [27]. This could potentially be explained by the gift exchange theory that involves the norm of reciprocity; reciprocity keeps continuous communication and health-related behavior among people [28]. Cigarette gifting could be seen as calculated reciprocity, for cigarettes are exchanged as gifts among adults and adolescents to show respect and appreciation in China, especially during holidays or important events (e.g., weddings). Reciprocity is a fundamental feature of social exchange [29]. Moreover, in many ways, cigarettes function as a “generalized reinforcer” that can be exchanged for social opportunities and benefits [23]. Based on behavioral consistency, we assume that the social exchange of these cigarette substitutes might also promote tobacco spread.

Considering the limited research on e-cigarette and tea cigarette gifting and the potential unknown adverse consequences of these gifting behaviors, the purposes of the current study are to (1) investigate e-cigarette and tea cigarette gifting and their influencing factors and (2) to explore the relations of e-cigarette and tea cigarette gifting behaviors with smoking and cessation in China.

## 2. Materials and Methods

### 2.1. Participants and Procedure

This study utilized a multistage sampling design. First, we selected two provinces in China and subsequently chose one university from each of the provinces based on their regional characteristics and existing research collaboration with the primary investigators. All classes that had health professional courses were selected from each university. Students who were enrolled in the chosen classes and came from the local province were selected. These students were then invited to distribute the survey link to their heads of household (HH). A total of 1240 HHs in Guangdong Province and 755 HHs in Shaanxi Province were recruited. Household heads who were born in the two selected provinces and whose place of residence in the last 12 months was also in that same province were included. Finally, 982 household heads from Guangdong Province and 530 household heads from Shaanxi province were included in the data analysis. More detailed information on the sampling and recruitment process can be found in Wu et al. (2022) [27] Our survey was completely anonymous, developed on the Wenjuanxing Platform (https://www.wjx.cn/app/survey.aspx, accessed on 30 April 2020), and conducted online from 30 April to 30 July 2020.

### 2.2. Measures

Demographic data included in the study were age, sex, marital status, place of residence, education, occupation, and annual household income. Participants were asked whether they were current smokers and how often they smoked. Daily smokers were those who smoked every day, and occasional smokers were those who occasionally smoked [30]. Participants were further asked what type of tobacco products or similar alternatives they smoked. The multiple-choice responses included e-cigarettes and tea cigarettes.

Cessation attempt was measured by asking, “have you ever had the experience of quitting smoking?” Participants answering “Yes” were categorized as “previous cessation attempters”. According to the above smoking and quitting questions, respondents who did not smoke currently and had successfully quit were classified as “former smokers” or “successful quitters”; respondents who smoked currently, but had a quit attempt experience, were classified as “unsuccessful quitters” or “fail quit attempt”.

Gifting cigarettes was defined as giving and receiving at least one unopened pack of 11 cigarettes [24,31]. It was measured by asking if the participants agreed with the following statements: “in the past year, have you given someone/received at least an e-cigarette or one unopened pack of tea cigarettes as a gift?” Response categories were ‘yes’ and ‘no’.

Social participation was measured by asking frequency of ‘meeting with others for food and drink’, ‘going to leisure activities organized by your local work unit or commune’, ‘volunteering for public causes’, and ‘meeting with family members for entertainment’ [32,33]. They were rated as: never, once a few months, once a month, 2–3 times per month, and ≥4 times per month. A total score was derived by summing the item scores. They were divided into a ‘high score’ group (>8.767) and a ‘low score’ group (≤8.767) according to the average score (8.767).

### 2.3. Data Analysis

Data were entered into Microsoft Excel and then imported into SPSS (V. 25.0) for statistical analyses. Descriptive statistics were calculated for the distribution of e-cigarette/tea cigarette gifting experience. We conducted Chi-square tests to determine differences between e-cigarette/tea cigarette gifting experience and demographic, socioeconomic characteristics, smoking status, and social participation. Logistic regression analysis was used to identify the influencing factors associated with e-cigarette/tea cigarette gifting (*p* < 0.05) and to identify whether e-cigarette/tea cigarette gifting was associated with tobacco use and cessation failure. It was performed using backward stepwise regression analysis. The variables that were statistically significant in univariate analysis were included in the regression model.

## 3. Results

Table 1 presents the socio-demographic profile of the participants. The average age of the participants was 47.8 (SD: 9.3) years. Among the sample, 22.2% were under 45 years old, and 39.2% were between 45 and 49 years old. Moreover, 82.5% were male, 88.2% were married, 47.3% grew up in rural areas, and 58.1% did not receive a high school education. The participants included managers/owners (2.1%), white-collar workers (16.1%), blue-collar workers (44.3%), and service-class workers (15.1%). Of the participants, 33.1% smoked every day and 8.8% used cigarettes occasionally. Among the 643 smokers, 2.2% (14) were e-cigarette users, 5.4% (34) were tea cigarette users, and 85.2% (540) were traditional cigarette users.

### 3.1. The Demographic Characteristics Associated with Gifting E-Cigarettes/Tea Cigarettes

Among the participants, 456 (30.2%) individuals had offered tea cigarettes, 474 (31.3%) received tea cigarettes, 36 individuals (2.4%) had offered e-cigarettes, and 48 (3.2%) received e-cigarettes. Table 1 shows the univariate analysis (Chi-square tests) for each demographic characteristic that might be associated with gifting e-cigarettes/tea cigarettes. Participants who were white-collar had a lower likelihood of receiving (OR = 0.10, *p* < 0.01) e-cigarettes compared to managers/owners. Those who were married were significantly more likely to offer (OR = 2.41, *p* < 0.01) and receive (OR = 1.88, *p* < 0.01) tea cigarettes. Those from Shannxi province had a higher likelihood of offering (OR = 3.91, *p* < 0.01)/receiving (OR = 4.16, *p* < 0.01) e-cigarettes and a lower likelihood of receiving (OR = 0.65, *p* < 0.05) tea cigarettes, in comparison with people from Guangdong province. Meanwhile, higher social participation had a higher likelihood of offering (OR = 2.51, *p* < 0.05)/receiving (OR = 2.23, *p* < 0.05) e-cigarettes and offering (OR = 1.44, *p* < 0.01)/receiving (OR = 1.46, *p* < 0.01) tea cigarettes (Table 2).

The results from multiple logistic regression showed that exposure to tobacco use was related to gifting e-cigarettes. Daily smokers were more likely to receive e-cigarettes (*OR* = 2.52, *p* < 0.05), and occasional smokers had a greater chance of offering (*OR* = 4.20, *p* < 0.01) and receiving (*OR* = 4.72, *p* < 0.01) e-cigarettes. Both daily smokers (OR = 1.57, *p* < 0.01) and occasional smokers (*OR* = 1.55, *p* < 0.05) were more likely to receive tea cigarettes than nonsmokers (Table 2).

### 3.2. Gifting E-Cigarettes/Tea Cigarettes Associated with Cigarette Smoking Behavior and Quitting Failure

We adjusted all demographic characteristics and social participation variables in the applications of models 1–2. The logistic regression analysis found that receiving e-cigarettes (*OR* = 3.43, *p* < 0.05) and tea cigarettes (*OR* = 1.70, *p* < 0.01) were related to tobacco use. Smokers who have received e-cigarettes (*OR* = 9.85, *p* < 0.01) and tea cigarettes (*OR* = 1.92, *p* < 0.05) were also less likely to quit smoking (Table 3).

## 4. Discussion

Our study showed that 2.2% of smokers used e-cigarettes, and 5.4% of smokers used tea cigarettes. More than 30% of participants have offered or received tea cigarettes, which was much more than those who had offered or received e-cigarettes (about 3%). Our previous research showed that, among the participants, 23.9% offered traditional cigarettes to others, while 18.5% reported receiving cigarettes as gifts from others in the past twelve months [27]. It is noted that the frequency of tea cigarette gifting is even higher than traditional cigarettes. This might be because some merchants claim that tea cigarettes are “healthier” than traditional cigarettes. Moreover, the packages of tea cigarettes always have symbols and idioms that connote happiness or prosperity, which could show respect and hospitality [34,35]. They are also similar to traditional cigarette packages, which could help them be accepted and gain a larger consumer base within a short time.

### 4.1. Sociodemographic Characteristics Associated with Gifting Alternative Cigarette Products Practices

The propensity to give e-cigarettes/tea cigarettes as gifts was found to be influenced by multiple factors. People who are married were more likely to offer tea cigarettes, and the middle or older age group was more likely to receive tea cigarettes as compared to young people. This could be because middle-aged and elderly people in China like to drink tea, and as such, it is easier for tea cigarettes to become popular among them. Previous research found that tobacco companies marketed and promoted their products to young people as they were more likely to accept new items [14]. Our results showed that newer and emerging tobacco products such as tea cigarettes might be targeted at the middle-aged and the old to keep smoking and contribute to their failed quit attempts. Managers/owners were more likely to receive e-cigarettes compared to white-collar workers, which might be due to their more frequent engagement in social activities. Our study also showed that provinces were associated with tea cigarettes and e-cigarette gifting. The difference in culture and economic status between northern and southern China may result in different customs of e-cigarette and tea cigarette gifting. Southern China is the hometown of tea, tea trees, tea drinking, and tea culture. Guangdong is one of the four tea culture centers in China. It is also the producer of tea brands that have been widely recognized and commercialized in south China [36]. Drinking tea also plays an important role in the social lives of Guangdong.

Our findings showed that daily smokers were more likely to receive e-cigarettes and tea cigarettes than nonsmokers, and occasional smokers had a greater chance of offering and receiving e-cigarettes. Although the new products do not taste like traditional cigarettes, e-cigarette and tea cigarette gifting is similar to traditional cigarette gifting [27,37]. Most participants who accepted the offered cigarettes were smokers [38]. It implies that e-cigarettes and tea cigarettes could become popular among smokers, particularly occasional smokers. It is likely that this type of smoker is more willing to try or share new products with others. Daily smokers were more likely to find a “healthier” replacement, such as a tea cigarette. Social participation was a prominent risk factor for e-cigarette and tea cigarette gifting. A person with a higher social participation score had a higher potential to offer or receive e-cigarettes and tea cigarettes. Cigarette gifting is a social epidemic that stems from cultures or social norms [23]. Gifting cigarette substitute products is also a social behavior [39,40]. The Chinese value works in the social networks called guanxi. Guanxi means ‘interdependent relationship’. Guanxi is also seen as a variant form of social participation [41]. Sharing cigarettes products provides a clear avenue to maintain and expand a person’s guanxi [27,28], and new tobacco products such as e-cigarettes and tea cigarettes could also be used to show respect or care for elders or friends who are smokers as they are perceived to be “healthier” than traditional cigarettes. Marital status, smoking status, provinces, and social participation were also significantly associated with gifting traditional cigarettes [27].

### 4.2. The Gifting Practice Association with Current Smoking and Failed Quit Attempt

To the best of our knowledge, this is one of the first studies to explore the relationship between gifting e-cigarettes and tea cigarettes with tobacco use. The result showed that people who receive e-cigarettes or tea cigarettes are at higher risk of tobacco use and cessation failure compared to those not receiving them. This could be explained by Behavioral Susceptibility Theory (BST) and Social Learning Theory (SLT).

BST argues that if a given behavior becomes convenient or easy, this behavior will gradually increase [42]. Receiving e-cigarettes or tea cigarettes and then being exposed to cigarette substitute products could be seen as a temptation situation that induces smoking behavior. SLT emphasizes learning by observing, imitating, and modeling. It also posits that smoking is a learned behavior acquired through social interactions and reinforcement [43].

### 4.3. Policy Implications

The regulatory mechanism for tea cigarettes is unclear, and their safety is difficult to guarantee. Further relevant research needs to be conducted regarding tea cigarettes, especially as it pertains to their health effects and whether they could help people who smoke quit. Studies using randomized designs should be conducted to assess the health risks and benefits of gifting cigarette alternatives such as e-cigarettes and tea cigarettes.

### 4.4. Study Limitations

There are several limitations to this study. First, this study utilizes a cross-sectional design, which prohibits causal inferences. Second, the findings cannot be generalized due to the use of convenient sampling. Third, the study was conducted in two provinces, which do not represent the entire population of China. Furthermore, selection bias might exist, and we did not collect sociodemographic information on non-respondents. Lastly, the participants in this study were household heads with an average age of 47.8 years old, and 83% were male, which means this study did not cover all groups, especially young people and females. While this limits generalizability, male household heads might be more likely to be involved in tobacco-related gifting behaviors. Given this, it is important to pay more attention to this issue among this population.

## 5. Conclusions

The propensity to gift e-cigarettes/tea cigarettes is associated with multiple factors. The differences in culture and economic status between provinces of northern and southern China may result in different customs of e-cigarette and tea cigarette gifting. Social participation was correlated with these gifting behaviors of cigarette replacements. Further, e-cigarette/tea cigarette gifting may further promote tobacco use and ultimately harm people’s health. Specifically, people who received e-cigarettes or tea cigarettes were related to tobacco use and cessation failure. Further, research on the benefits and risks of gifting e-cigarettes and tea cigarettes is warranted. The findings from this study also suggest that the government should pay attention to tea cigarettes and their health effect on human beings.

## Figures and Tables

**Table 1:** Demographic characteristics of the sample and the distribution of e-/tea cigarette gifting.

Variables	n (%)	Gifting E-Cigarettes				Gifting Tea Cigarettes			
		Offering		Receiving		Offering		Receiving	
		n (%)	*p*	n (%)	*p*	n (%)	*p*	n (%)	*p*
		36 (2.4)		48 (3.2)		456 (30.2)		474 (31.3)	
Age			0.126		0.081		0.175		0.008 **
<45	336 (22.2)	13 (3.9)		17 (5.1)		90 (26.8)		82 (24.4)	
45–49	593 (39.2)	12 (2.0)		15 (2.5)		193 (32.5)		196 (33.1)	
50+	583 (38.6)	11 (1.9)		16 (2.7)		173 (29.7)		196 (33.6)	
Gender			0.568		0.358		0.021 *		0.004 **
Male	1248 (82.5)	31 (2.5)		42 (3.4)		392 (31.4)		411 (32.9)	
Female	264 (17.5)	5 (1.9)		6 (2.3)		64 (24.2)		63 (23.9)	
Marital status			0.049 *		0.048 *		<0.001 **		0.001 **
Married	1334 (88.2)	28 (2.1)		38 (2.8)		426 (31.9)		439 (32.9)	
Unmarried	178 (11.8)	8 (4.5)		10 (5.6)		30 (16.9)		35 (19.7)	
Place of residence			0.742		0.929		0.683		0.326
Rural area	715 (47.3)	18 (2.5)		23 (3.2)		212 (29.7)		233 (32.6)	
Urban area	797 (52.7)	18 (2.3)		25 (3.1)		244 (30.6)		241 (30.2)	
Education			0.354		0.635		0.022 *		0.015 *
Elementary school or less	282 (18.7)	6 (2.1)		8 (2.8)		80 (28.4)		85 (30.1)	
Junior high school	595 (39.4)	19 (3.2)		22 (3.7)		201 (33.8)		214 (36.0)	
High school	353 (23.3)	5 (1.4)		8 (2.3)		108 (30.6)		100 (28.3)	
Junior college, college, or higher	282 (18.7)	6 (2.1)		10 (3.5)		67 (23.8)		75 (26.6)	
Occupation			0.066		0.001 **		0.083		0.054
Manager/Owner	31 (2.1)	3 (9.7)		4 (12.9)		16 (51.6)		17 (54.8)	
White-collar	244 (16.1)	4 (1.6)		3 (1.2)		68 (27.9)		72 (29.5)	
Blue-collar	670 (44.3)	7 (3.1)		13 (5.7)		72 (31.4)		70 (30.6)	
Service class	229 (15.1)	13 (1.9)		21 (3.1)		194 (29.0)		216 (32.2)	
Irregular employee	338 (22.4)	9 (2.7)		7 (2.1)		106 (31.4)		99 (29.3)	
Household annual income (yuan)			0.618		0.968		0.207		0.037 *
<20,000	494 (32.7)	10 (2.0)		15 (3.0)		137 (27.7)		143 (28.9)	
20,000–49,999	479 (31.7)	12 (2.5)		15 (3.1)		150 (31.3)		149 (31.1)	
50,000–79,999	208 (13.8)	6 (2.9)		7 (3.4)		56 (26.9)		56 (26.9)	
80,000–99,999	122 (8.1)	1 (0.8)		3 (2.5)		45 (36.9)		47 (38.5)	
100,000+	209 (13.8)	7 (3.3)		8 (3.8)		68 (32.5)		79 (37.8)	
Province			<0.001 **		0.001 **		0.027 *		0.002 **
Guangdong	982 (64.9)	10 (1.0)		14 (1.4)		315 (32.1)		334 (34.0)	
Shaanxi	530 (35.1)	26 (4.9)		34 (6.4)		141 (26.6)		140 (26.4)	
Smoking status			<0.001 **		0.001 **		0.143		0.002 **
Daily smoker	501 (33.1)	14 (2.8)		22 (4.4)		162 (32.3)		183 (36.5)	
Occasional smoker	133 (8.8)	11 (8.3)		14 (10.5)		46 (34.6)		47 (35.3)	
Nonsmoker	878 (58.1)	11 (1.3)		12 (1.4)		248 (28.2)		244 (27.8)	
Social participation			0.004 **		0.004 **		0.003 **		0.002 **
High score	696 (46.0)	25 (3.6)		32 (4.6)		236 (33.9)		246 (35.3)	
Low score	816 (54.0)	11 (1.3)		16 (2.0)		220 (27.0)		228 (27.9)	

* *p* < 0.05; ** *p* < 0.01.

**Table 2:** Logistic regression results of socio-demographic factors associated with gifting e-cigarettes and tea cigarettes.

	Gifting E-Cigarettes Odds Ratio (95%C. I.)		Gifting Tea Cigarettes Odds Ratio (95%C. I.)	
	Offering	Receiving	Offering	Receiving
Marital status	/	/		
Married	/	/	2.41 (1.60–3.63) **	1.88 (1.27–2.79) **
Unmarried	/	/	1.00	1.00
Occupation	/	/	/	/
Manager/Owner	/	1.00	/	/
White-collar	/	0.10 (0.02–0.52) **	/	/
Blue-collar	/	0.78 (0.22–2.82)	/	/
Service class	/	0.40 (0.12–1.37)	/	/
Irregular employed	/	0.30 (0.08–1.18)	/	/
Province	/		/	/
Shaanxi	3.91 (1.83–8.38) **	4.16 (2.13–8.11) **	/	0.65 (0.51–0.83) *
Guangdong	1.00	1.00	/	1.00
Smoking status			/	
Daily smoker	1.89 (0.84–4.25)	2.52 (1.21–5.25) *	/	1.57 (1.24–2.00) **
Occasional smoker	4.20 (1.72–10.27) **	4.72 (2.03–10.97) **	/	1.55 (1.04–2.32) *
Nonsmoker	1.00	1.00	/	1.00
Social participation			/	
High score	2.51 (1.21–5.20) *	2.23 (1.17–4.28) *	1.44 (1.15–1.80) **	1.46 (1.17–1.82) **
Low score	1.00	1.00	1.00	1.00

* *p* < 0.05; ** *p* < 0.01. / The variables that were not significant in univariate analysis have been excluded from the regression model.

**Table 3:** Logistic regression analysis for smoking and quitting situation with gifting e-cigarettes and tea cigarettes.

	Model 1#: Current Smoking Status among All Participants (N = 1512)	Model 2#: Quitting Failure Status among Previous Cessation Attempter (n = 571)
Gifting e-cigarettes		
Offering		
Yes	1.09 (0.37–3.16)	0.90 (0.21–3.97)
No	1.00	1.00
Receiving		
Yes	3.43 (1.30–9.09) *	9.85 (1.11–87.21) **
No	1.00	1.00
Gifting tea cigarettes		
Offering		
Yes	0.76 (0.53–1.10)	0.59 (0.31–1.10)
No	1.00	1.00
Receiving		
Yes	1.70 (1.19–2.42) **	1.92 (1.03–3.57) *
No	1.00	1.00

* *p* < 0.05; ** *p* < 0.01. All demographic characteristics and social participation were adjusted.

## Data Availability

The datasets used in the current study are available from the corresponding author upon reasonable request.
